# Methyl Gallate from *Galla rhois* Successfully Controls Clinical Isolates of *Salmonella* Infection in Both *In Vitro* and *In Vivo* Systems

**DOI:** 10.1371/journal.pone.0102697

**Published:** 2014-07-21

**Authors:** Jang-Gi Choi, Su-Hyun Mun, Harendra S. Chahar, Preeti Bharaj, Ok-Hwa Kang, Se-Gun Kim, Dong-Won Shin, Dong-Yeul Kwon

**Affiliations:** 1 Department of Oriental Pharmacy, College of Pharmacy, Wonkwang University, Wonkwang Oriental Medicines Research Institute, Institute of Biotechnology, Jeonbuk, Korea; 2 Center of Excellence in Infectious Disease Research, Department of Biomedical Sciences, Paul L. Foster School of Medicine, Texas Tech University Health Sciences Center, El Paso, Texas, United States of America; 3 BK21 Plus Team, Professional Graduate School of Oriental Medicine, Wonkwang University, Iksan, Jeonbuk, Republic of Korea; 4 Department of Oriental Medicine Resources, Sunchon National University, Jeonnam, Republic of Korea; Albert Einstein College of Medicine, United States of America

## Abstract

*Galla rhois* is a commonly used traditional medicine for the treatment of pathogenic bacteria in Korea as well as in other parts of Asia. Methyl gallate (MG), a major component of *Galla Rhois*, exhibits strong antibacterial activity, but its mechanism of action against *Salmonella spp*. is unclear. In the present study, we investigated the antibacterial actions of MG against *Salmonella*. The antibacterial activity determined by broth dilution method indicated that the antibacterial activity of MG against *Salmonella* strains ranged from 3.9 to 125 µg/ml. *In vitro* bacterial viability test indicated that MG significantly decreased the viability of *Salmonella* over 40% when combined with ATPase inhibitors. The time-kill curves showed that a combined MG and ATPase inhibitors (DCCD and NaN3) treatment reduced the bacterial counts dramatically after 24 h. Oral administration of MG showed a strong anti-bacterial activity against WS-5 infected BALB/c mice. In contrast to the untreated *Salmonella* infected control animals, MG treated groups showed no clinical symptoms of the disease, such as lethargy and liver damage. It was observed that MG treatment significantly increased the survival of animals from *Salmonella* infection, while in untreated groups all animal succumbed to disease by the sixth day post infection. Thus, the present study demonstrates the therapeutic ability of MG against *Salmonella* infections.

## Introduction

*Salmonella* enterica is a gram-negative bacterial pathogen capable of infecting animals and humans, causing significant morbidity and mortality worldwide [1]. *Salmonella* is a clinically important intracellular bacterial pathogen that leads to food poisoning and gastroenteritis in millions of people worldwide each year [2]. It is even a problem in industrialized nations, and the Centers for Disease Control (CDC) estimate that there are nearly 1.4 million foodborne *Salmonella* infections annually in the United States [3]. *S.* Typhimurium, *S.* Enteritidis and *S.* Typhi were the most frequent *Salmonella* serovars in foodborne diseases and diarrhea patients in Korea from 1998 to 2007 [4]. Similarly, serotypes that are highly adapted to poultry include *S*. Gallinarum, resulting in high morbidity and mortality [5].

Following oral acquisition, *Salmonella* infects the intestinal tract and can disseminate to cause systemic infection of various organs including the liver [6]. One major concern to public health has been the global dissemination of *Salmonella* Typhimurium definitive Type 104, which is commonly resistant to five or more antimicrobial agents [7]–[10]. Recent reports of infections related to strains of *Salmonella* with high-level resistance to antibiotics are therefore particularly worrying. The rise in antibiotic-resistant pathogens has led to the development of new therapeutic agents that are effective against these bacteria. Recently, there has been considerable interest in the use of plant materials as an alternative method to control pathogenic microorganisms [11], and many compounds of plant products have been shown to specifically target against resistant pathogenic bacteria [11].

*Galla rhois* is the term used for the gall caused by the Chinese sumac aphid, *Schlechtendalia chinensis* (Bell), on the nutgall sumac tree, *Rhus javanica* L. (Anacardiaceae) [12]. It has been used in traditional Korean medicine and other oriental medicine systems for years. It has long been used for the treatment of diarrhea, prolonged coughing, and spontaneous perspiration in Korea. It is a natural non-toxic traditional Korean medicine and contains several tannin-derived components, such as methyl gallate (MG) and gallic acid [12]. MG is a phytochemical compound with strong antioxidant properties found in various species including Meliaceae, *Galla Rhois*, *Rosa Rugosa* and it is also well known as an anti-oxidative beverage [13]. In addition, MG possesses other biological activities such as anti-platelet activity, protection of DNA damage against oxidative stress [14], protection of lung injury induced by phosgene [15], attenuation of diabetic oxidative stress and anti-apoptotic activity [15]. MG is known to perform a wide spectrum of biological activities, but its mechanism for antibacterial activity against *Salmonella* remains unclear. We hypothesized that there is synergy between MG and bacterial membrane permeablization/binding agents such as tris(hydroxymethyl)aminomethane (TRIS), triton X-100 (TX) [16]–[18]. MG may be responsible for its increased antibacterial activity against *Salmonella*. Second, MG may exert its inhibitory effect on the bacterial replication by inhibiting cytochrome oxidase or by inhibition of proton-driven ATPases [16]–[18]. As a result, we decided to investigate the *in vitro* activities of MG separately or in combination with the bacterial membrane- binding agents TRIS and TX, and the ABC transporter-inhibiting agents NaN3 and inhibitor of proton-driven ATPases *N,N′*-Dicyclohexylcarbodiimide (DCCD). In addition, we investigated the antimicrobial activity of MG against *Salmonella in vivo*.

## Materials and Methods

### Plant materials

*Galla Rhois*, purchased from the Oriental drug store Daehak Hanyak kuk (Iksan, Korea), was authenticated by Dr. D.Y. Kwon. A voucher specimen (No. 06-021) was deposited in the Laboratory of Herbalogy, College of Pharmacy, Wonkwang University, Iksan, Korea.

### Bacterial strains

Various *Salmonella* listed in Tables S1, were used in this study. We also tested the antimicrobial activity of MG against local isolates of *S*. Enteritidis, *S.* Gallinarum and *S.* Typhimurium, which were generously provided by the National Veterinary Research and Quarantine Service, Republic of Korea and kept as frozen glycerol stock. Cells in frozen stock were streaked onto nutrient agar medium to produce cell colonies, from which a single colony was transferred to Mueller-Hinton agar (MHA). For preparation of inocula, cells were grown for 20 h at 37°C in Mueller-Hinton broth (MHB). For mouse infection, cultured bacterial cells (WS-5) were recovered by centrifugation at 13,000 rpm for 30 s and then washed with and resuspended in PBS [19]. The McFarland standard turbidity of the cell suspensions was measured. The cell suspensions were diluted with PBS to the desired concentration of bacteria using a standard curve of optical density versus bacterial number determined as colony-forming units (CFU). Confirmation of *Salmonella* strains were performed by a PCR assay described by a previous report [20], [21].

### Reagents and instruments

NMR spectra were measured with a JEOL Eclipse 500 FT-NMR spectrometer (^1^H, 500 MHz; ^13^C 125 MHz). Column chromatography was carried on silica-gel (Kieselgel 60, 70–230 mesh, Merck, Germany), a thin layer chromatography (TLC) on pre-coated Silica-gel F254 (0.25 mm, Merck) and Sephadex LH-20 (25–100 µM, Sigma, U.S.A). Mueller-Hinton broth (MHB) and Mueller-Hinton agar (MHA) (Difco Laboratories, Baltimore, MD, USA). Ampicillin, amoxicillin/clavulanic acid, chloramphenicol, cephalothin, sulfisoxazole, nalidixic acid, norfloxacin, streptomycin, trimethoprim/sulfamethoxazole, ticarcillin, ciprofloxacin, *N,N′*-Dicyclohexylcarbodiimide, Sodium azide, Tris, Triton X-100 and solvents were purchased from Sigma Aldrich (St. Louis, USA).

### Isolation of methyl gallate

**Figure 1:**
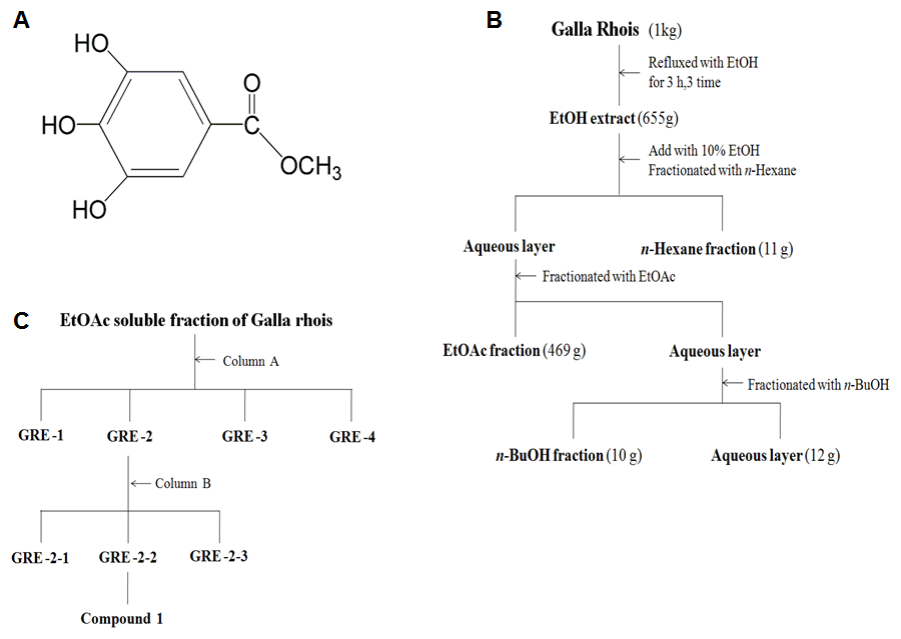
Isolation of methyl gallate from *Galla rhois*. (A) Chemical structure of methyl gallate, (B) Procedures of extraction and fraction of the *Galla Rhois*, (C) Column chromatographic procedures of n-EtOAc fraction.

The EtOH extracts were partitioned with organic solvents of different polarities to yield n-Hexane, EtOAc, n-BuOH and H_2_O fractions, in sequence (Figure 1). The EtOAc fraction of each plant was subjected to silica gel chromatography with CH_2_Cl_2_-MeOH-H_2_O (lower layers, by volume, 5∶1∶1→7∶3∶1) as the solvents to yield MG from *Galla Rhois* (Figure 1). The structure of the compound (Table 1) was determined by its physico-chemical and spectral data (1H-NMR and 13C-NMR) which were in agreement with those reported in literature [22], [23].

**Table 1:** ^1^H- and ^13^C-NMR spectral data of compound 1 (methyl gallate) isolated from *Galla Rhois*.

Carbon NO.	^1^H-NMR	^13^C-NMR
1		119.8
2	6.93	109.1
3		146.1
4		138.9
5		146.1
6	6.93	109.1
COOCH_3_		166.8
COOCH_3_	3.73	52.1

Solvent: DMSO-*d_6_*.

### Antimicrobial resistance testing

Resistance of the *Salmonella* strains to the different antimicrobial agents was determined using the disc-agar method based on interpretations according to Clinical and Laboratory Standards Institute (CLSI) [24]. The quality control strains were *Enterococcus faecalis* ATCC 29212 and *Escherichia coli* ATCC 25922 (Table S1).

### Disc diffusion

The paper disc diffusion method was used to determine anti-bacterial activity [25]. Sterile paper discs (6 mm; Toyo Roshi Kaihsa, Japan) were loaded with 20 µl of MG (varying concentrations: 10, 50, and 100 µg) dissolved in 10% dimethyl sulfoxide (DMSO, Sigma, USA), and were left to dry for 12 h at 37°C in a sterile room. The bacterial suspensions were diluted to match the 0.5 McFarland standard scale (approximately 1.5×10^8^ CFU/ml), and were further diluted to obtain the final inoculum. The MHA was poured into petri dishes and inoculated with 100 µl of the suspension containing 1×10^5^ CFU of bacteria. The inhibition zone diameter around each of the discs was measured and recorded at the end of the incubation period. Ampicillin was included as positive control and 10% DMSO served as negative controls.

### Determination of the minimum inhibitory concentrations (MICs)

The minimal inhibition concentration (MIC) values were determined for the microorganisms we found to be sensitive to MG during the disc diffusion assay. A preparation of the microorganism inocula was from 12 h broth cultures and the suspensions were adjusted to a 0.5 McFarland standard turbidity. Susceptibility tests were carried out by the standard broth micro dilution method [26] in MHB with an inoculum of approximately 5×10^4^ CFU/ml. The MHB was supplemented with serial MG at concentrations from 0.97 to 1000 µg/ml. The data were reported as MICs, the lowest concentration of MG inhibiting visible growth after 24 hours of incubation at 37°C [26]. The MICs of ampicillin was also determined, and similarly defined as the lowest antibiotic concentration at which no visible bacterial growth was observed.

### Anti-bacterial activity with detergents or ATPase-inhibitors

To elucidate whether anti-bacterial activity of MG was associated with the altered membrane permeability or anti-bacterial susceptibility, effect of MG was examined in the presence of detergents or ATPase-inhibiting agents. Minimum inhibitory concentrations (MICs) of the selected antimicrobial agents (detergents or ATPase-inhibiting agents) were determined by micro broth dilution assays. To increase the permeability of the outer membrane, the concentration of MG, (a fractional inhibitory concentration (FIC) determined in a combination assay with other therapeutic agents) was added to bacterial cells in the presence of 0.001% Triton X-100, and 125 µg/ml Tris, respectively. NaN3 and DCCD were used as inhibitors of ATPase [16]–[18]. The anti-bacterial susceptibility of MG in the presence of 0.005% NaN3 and 125 µg/ml DCCD, was also carried out at the same condition. The viability of bacteria was determined by a spectrophotometer (optical density at 600 nm, OD_600_) after incubation for 24 h. *In vitro* synergy between the agents was evaluated by time-kill assay as described previously [18]. The synergy between each pair of antimicrobial agents was determined using time-kill curves of bacterial growth in 96-well plates at five different time-points (0, 2, 4, 8 and 24 h) (12). Bacterial cultures were diluted with fresh MHB to ∼1.5×10^5^ CFU/ml, and incubated at 37°C for 24 h. Aliquots (0.1 ml) of the culture were taken following 0, 2, 4, 8 and 24 h of incubation, and serial 10-fold dilutions were prepared in saline. For samples obtained from each time-point, the number of viable cells was determined on a drug-free MHA plate following incubation for 24 h. Colony counts were performed on plates and 30–300 colonies were counted. The lower limit of sensitivity for the colony counts was 100 CFU/ml. Antimicrobial agents were considered to be bactericidal at the lowest concentration that reduced the original inoculum by 3 log10 CFU/ml (99.9%) for each of the indicated time-points [22]. Synergy was defined as ≥2 log10 decrease in the number of CFU/ml between the combination and the most active compound [27], [28]. To confirm the results, time-kill assays for each experiment were performed in triplicate. Data are presented as the mean ± standard deviation [18].

### *In vivo* assay using mice

Mice were obtained from Da Mool Science (Daejeon, Korea). All mice experiments in this study were approved by the Wonkwang National University Animal Ethics Committee in accordance with the guidelines of the Korean Council on Animal Care. Thirty male BALB/c mice (15∼17 g) between 5 and 6 weeks old were used for all *in vivo* experiments. All animals were kept in a temperature-controlled room under a 12 h light ∼12 h dark cycle. Animals had free access to commercial solid food (SCF Co., Ltd. Korea) and water ad libitum, and were acclimatized for at least 1 week prior to beginning the experiments. Mice were divided into the following groups: *Salmonella*-infected (SI), and *Salmonella*-infected + MG (SIMG). For survival and fecal shedding each treatment group contained 10 mice in SI and SIMG group. Each mouse was caged individually and subjected to infection as described below. For histopathologic examinations, a separate set of 5 mice per group (SI and SIMG) were infected and sacrificed consecutively for 3 days post infection. Tissue specimens of the liver were transferred into 10% buffered neutral formalin for histopathologic examinations and then processed using standard procedures. Sections of paraffin-embedded tissues were then stained with hematoxylin and eosin. Throughout each experiment, mice were provided with water that contained streptomycin (5 mg/ml) to reduce the level of facultative anaerobic bacteria that normally colonize the mouse intestine [28]–[30]. *S.* Typhimurium (WS-5) was grown overnight in Luria–Bertani broth (Difco), centrifuged, washed in phosphate-buffered saline (PBS), and then diluted to achieve a final concentration of 1×10^4^ CFU. Mice were orally infected with *S.* Typhimurium (1×10^4^ CFU) using a gavage needle; the suspensions were then diluted in 20% sucrose and fed to the SI and SIMG groups. One hour after infection, animals in the SIMG group were orally administered 50 mg/kg of the MG daily by gavage needle as described before [28], SI group treated with sterile PBS and administered to the animals in similar manner. Fecal samples were collected at 0, 1, 3, 4 and 5 days after administering the bacterial suspensions and the numbers of the bacteria per gram in feces were determined. Aliquots (100 µl) of fecal suspensions were serially diluted in PBS and then were plated on duplicate Salmonella–Shigella agar plates (Difco), which were then incubated overnight at 37°C, and typical colonies were counted for plates containing between 30 and 300 colonies. Confirmation of *S*. Typhimurium was performed by a PCR assay described by a previous report [20], [21].

### Statistical analysis

The data were analyzed using Graph-Pad Prism software 5 (GraphPad Software, Inc., San Diego, CA) or Microsoft Excel. Results are given as means with standard deviation. Comparisons were made using the Pearson two-tailed test. All data with P<0.05 were considered significant.

## Results

### Anti-bacterial activity of MG against *Salmonella*

The antimicrobial efficacy of MG against the ten *Salmonella* strains was evaluated by the disc diffusion method via determination of the surrounding inhibition zones, as well as by evaluating the MIC using the broth micro dilution method. Table 2 shows the antimicrobial activity of MG determined by the disc diffusion method. The values of the inhibition zones produced against the tested bacteria ranged between 12 and 26 mm. The growth of all the tested strains was inhibited at 100 µg per disc. In addition, the response was dose-dependent, meaning the higher the dose the wider the inhibition zone. The MICs for MG and ampicillin against the 10 strains of *Salmonella* are shown in Table 3. The MICs determined using the broth dilution method confirmed the antimicrobial effects found through the disc diffusion method. MG showed antimicrobial activity against all the tested strains. The MICs of MG against the 10 *Salmonella* strains ranged from 3.9 to125 µg/ml, and for ampicillin from 0.09 to 1000 µg/ml.

**Table 2:** Antimicrobial activity (as the inhibition zone diameter) of methyl gallate (MG) and ampicillin against *Salmonella*.

Strains	Serotypes	Origin	MG			^a^Ampicillin
			10 µg	50 µg	100 µg	10 µg
WS- 1	*S.* Gallinarum ATCC 9184	Chicken	*ND	11	20	ND
WS- 2	*S.* Gallinarum	Chicken	ND	ND	15	ND
WS- 3	*S.* Gallinarum	Chicken	ND	ND	14	27
WS- 4	*S.* Typhimurium	Cattle	ND	ND	12	30
WS- 5	*S.* Typhimurium	Pig	ND	7	12	ND
WS- 6	*S*. Enteritidis	Human	7	18	25	27
WS- 7	*S*. Typhi ATCC 19943	Human	8	15	23	30
WS- 8	*S*. Paratyphi A	Chicken	7	14	26	28
WS- 9	*S.* Enteritidis	Chicken	ND	13	20	33
WS- 10	*S.* Enteritidis	Chicken	ND	10	13	31

*ND, No activity detected, ^a^ Positive control.

**Table 3:** Antimicrobial activity of MG and ampicillin against 10 strains of *Salmonella*.

Strains	Serotypes	Origin	aMIC (ug/ml)	
			MG	^b^Ampicillin
WS- 1	*S.* Gallinarum ATCC 9184	Chicken	31.25	1000
WS- 2	*S.* Gallinarum	Chicken	3.9	1000
WS- 3	*S.* Gallinarum	Chicken	15.6	0.97
WS- 4	*S.* Typhimurium	Cattle	3.9	0.97
WS- 5	*S.* Typhimurium	Pig	15.6	1000
WS- 6	*S.* Enteritidis	Human	3.9	1.95
WS- 7	*S.* Typhi ATCC 19943	Human	3.9	0.97
WS- 8	*S.* Paratyphi A	Chicken	31.25	0.97
WS- 9	*S.* Enteritidis	Chicken	125	0.97
WS- 10	*S.* Enteritidis	Chicken	31.25	1.95

a MIC, Minimum inhibitory concentration. ^b^ Positive control.

### Anti-bacterial activity with detergents or ATPase-inhibitors

**Figure 2:**
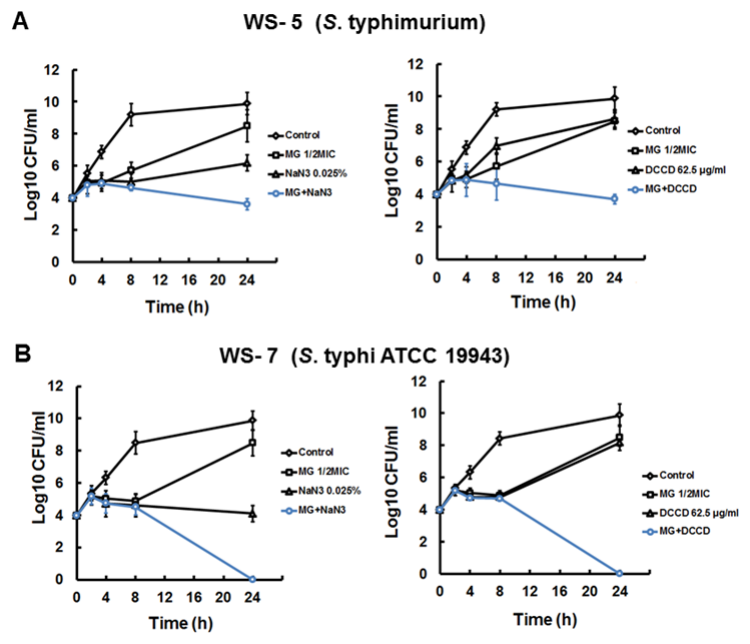
Time-kill curves for (A) *S*. Typhimurium (WS-5) and (B) *S*. Typhi ATCC 19943 (WS-7) with MG and ATPase inhibitors (DCCD and NaN3). These results were confirmed in three independent experiments.

To investigate the effects of enhanced membrane permeability on the activity of MG using detergents, the anti-bacterial activity of MG under increased membrane permeability was examined using 125 µg/ml Tris, and 0.001% Triton X-100. Tris and Triton X-100 all of which are membrane-permeabilizing agents (all reagents used 1/2 MIC) that can increase the permeability of the outer membrane in Gram negative bacteria by binding lipopolysaccharide (LPS) [31], [32]. These agents did not have any enhancing effect on the antibacterial activity of MG (Figure S1). We investigated bacterial viability in the presence of MG with 0.005% NaN3 and 125 µg/ml DCCD (all reagents used 1/2 MIC) as a metabolic inhibitor which can decrease ATP levels by disrupting electrochemical proton gradients in a bacterial environment [33], [34]. MG in combination with NaN3 and DCCD significantly decreased the viability of WS-5 over 40% (Figure S1). Time–kill assays were performed for MG in combination with ATPase inhibitors (NaN3 and DCCD) for two strains *S*. Typhimurium (WS-5) and *S*. Typhi ATCC 19943 (WS-7) strains. Figure 2A and B shows that MG and DCCD alone at 1/2 MIC had very week effect against both stationary-phase WS-5 and WS-7. NaN3 alone at 1/2 MIC reduced the CFU counts 3∼5 log of bacteria 24 h of incubation. However, the combination of MG and DCCD or NaN3 reduced the CFU counts to zero at 24 h of incubation for WS-7 and also MG and DCCD or NaN3 reduced CFU counts 2∼4 log the period of 24 h of incubation for WS-5. The time–kill assay demonstrated that there was a significant synergistic activity between MG and NaN3 or DCCD for the *Salmonella* strains tested (Figure 2).

### *In vivo* assay using mice

**Figure 3:**
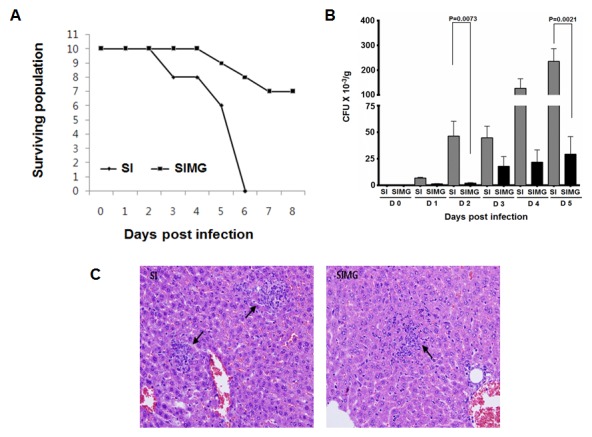
Therapeutic effects of MG treatment on mice infected with *Salmonella*. (A) the survival of mice (n = 10, per group) treated or untreated with MG, infected with WS- 5, (B) Effects of feeding MG on fecal shedding of *S*. Typhimurium (CFU/g) from mice. (C) Histopathological changes in liver of *Salmonella*-infected and *Salmonella*-infected treated MG. *Salmonella*-infected (SI) and *Salmonella*-infected + MG (SIMG). All data with P<0.05 were considered significant.

Barthel *et al.* [30] described a versatile animal model to understand the molecular mechanisms of enteric salmonellosis. We employed a similar *in vivo* model in the present study to determine the protective effects of MG against *S.* Typhimurium (WS- 5) infection in mice. Briefly, mice were infected with 1×10^4^ CFU of *S.* Typhimurium (SI). One hour later, the mice were orally administered MG (50 mg/kg). As shown in Figure 3A, treatment with the MG was found to have marked effects on mortality and in the MG treated group 70% of the animals survived while all animals in the untreated group infected with *Salmonella* died by the sixth day post infection. MG was found to have marked effects on the numbers of viable *S*. Typhimurium recovered from feces. At day 3 post-infection, seven of ten mice in the test group did not shed viable *S.* Typhimurium in feces, whereas all mice (1 mouse died) in the control group shed bacteria ranging 1.7×10^3^∼1.2×10^5^ CFU/g in feces (Figure 3B). *S.* Typhimurium-infected mice that did not receive the MG were lethargic and showed signs of histological damage in the liver. In addition, the central and portal veins of the liver showed congestion with focal necrotic emboi-like materials (Figure 3C). Five different sections from liver of each mice were tested for necrotic lesions and neutrophil expression. There were multiple small necrotizing nodular lesions in the liver parenchyma with inflammatory cell infiltrate. Conversely, clinical signs and histological damage were rarely observed in *S.* Typhimurium-infected mice fed with MG.

## Discussion

Due to the recent appearance of the “Super Bacteria” of *Salmonella* DT 104 showing resistance to multiple antibiotics and the intractable bacterial types of *Escherichia coli* (E. coli O157, O111, O26) releasing stronger toxin due to the application of antibiotics, the development of new antibiotics is urgently required, which is even tendered as a social issue [35], [36]. To overcome the emerging problem of bacterial antibiotic resistance, studies investigating plant extracts with antibiotics against clinical strains have been reported [7]–[9].

*Galla Rhois* is naturally found in Korea and China, where it is known as Chinese Sumac and has been used in the treatment of cold, fever, cough and malaria. It was previously reported that the methanol extract of *Galla Rhois* had significant growth-inhibitory activity towards both *Clostridium perfringens* and *Escherichia colis* [12]. MG is the main component of *Galla Rhois*, displaying several biological activities, and presents activity against *Salmonella*. It is observed that the anti-bacterial activity of MG described in this study is in agreement with results reported by others, showing that the activity of *Galla Rhois*, and of other species against *Salmonella*, is due to this compound [37], [38]. The potential of MG to enhance the activity of antibiotics against *Salmonella* was studied previously [37], [38] but the function of MG in *Salmonella* is still unclear. Therefore we investigated for the mechanism of MG against *Salmonella*. Antibacterial agents such as β-lactams antibiotics are inhibitor of cell wall synthesis [39], and fluoroquinolone antibiotics are inhibitor of DNA gyrase against both Gram-positive and Gram-negative bacteria [40], In this study, MG was used in combination with detergents (TX and Tris) responsible for increasing the membrane permeability of bacterial strain. However, additional decrease in bacterial replication was not observed as determined by the OD value, where the bacterial culture was treated with MG along with cell of membrane-permeabilizing agents. Which indicates that may be MG does not interfere with cell wall synthesis. In contrast, MG in combination with ATPase inhibitors (NaN3 and DCCD) significantly decreased the viability of *Salmonella* WS-5 as determined by spectrophotometry. As determined in time-kill studies, MG in combination with ATPase inhibitors shows synergistic effect against WS-5 and WS-7.

This finding indicates that the primary mechanism of MG action is via DNA gyrase or ATPase inhibition in the *Salmonella* but not via cell wall synthesis inhibition. In our previous study, we found that the combination of MG, at sub-MIC concentrations, with ciprofloxacin and nalidixic acid significantly improved the activity of the antibiotic [37], [38]. MG and fluoroquinolone antibiotics (ciprofloxacin and nalidixic acid) directly or indirectly attack the same target: DNA gyrase or ATPase inhibition in the *Salmonella* [39], [41]. The additive or neutral effects of MG in combination with other agents strongly support this explanation. In the *in vivo* experiments using mice, the test group dosed with 50 mg/kg of MG, showed clear MG mediated protection which resulted in survival of 70% of mice during 6 days of experiment. However, all the mice in SI group died by 6 day of the experiment. In addition, the cytological identification of liver in MG dosed mice revealed less expression of neutrophil in SIMG group as compared to SI group by showing the treatment effect of MG against murine salmonellosis.

Hence the findings of our study clearly demonstrate the usefulness of MG in treatment of *Salmonella* infections and may provide the preliminary supportive data for in-depth studies regarding the mechanism of action of MG against bacterial pathogens. Therefore, it is possible to conclude that MG has the potential for use in the treatment of *Salmonella* infections in mice.

## Supporting Information

Figure S1 **The effects of membrane-permeabilizing agent and ATPase-inhibitor agent on** ***Salmonella*** **(WS-5) susceptibility to MG.** The viability of bacteria was determined by a spectrophotometer (optical density at 600 nm, OD_600_) after incubation for 24 h with 1/2 MIC MG and the indicated concentration of Tris, TX, NaN_3_ and DCCD in WS-5 the data are Mean±S.D. for triple-independent experiments. A(Tris), B (TX), C(NaN_3_), D(DCCD).
(TIF)

Table S1 List of *Salmonella* strains used in this study and growth inhibition zones produced by antibiotics.
(DOCX)
