# Supplementary figures and images for: Methyl Gallate from Galla rhois Successfully Controls Clinical Isolates of Salmonella Infection in Both In Vitro and In Vivo Systems
Source: PLoS One. 2014 Jul 21;9(7):e102697. doi: 10.1371/journal.pone.0102697 (PMC4105534; doi:10.1371/journal.pone.0102697)

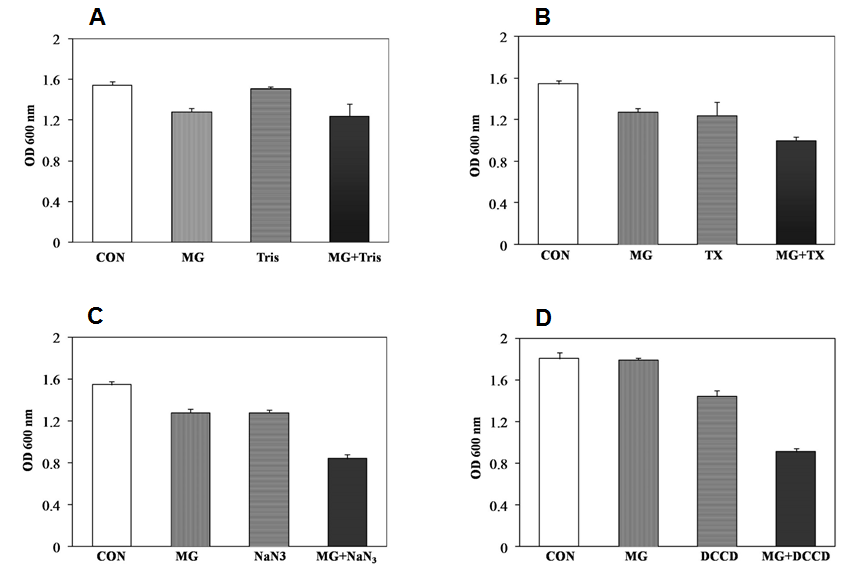

Supplement: Figure S1 — The effects of membrane-permeabilizing agent and ATPase-inhibitor agent on Salmonella (WS-5) susceptibility to MG. The viability of bacteria was determined by a spectrophotometer (optical density at 600 nm, OD600) after incubation for 24 h with 1/2 MIC MG and the indicated concentration of Tris, TX, NaN3 and DCCD in WS-5 the data are Mean±S.D. for triple-independent experiments. A(Tris), B (TX), C(NaN3), D(DCCD). (TIF) [file pone.0102697.s001.tif]
