# Supplementary material for: Methyl Gallate from Galla rhois Successfully Controls Clinical Isolates of Salmonella Infection in Both In Vitro and In Vivo Systems
Source: PLoS One. 2014 Jul 21;9(7):e102697. doi: 10.1371/journal.pone.0102697 (PMC4105534; doi:10.1371/journal.pone.0102697)
Supplement: Table S1 — List of Salmonella strains used in this study and growth inhibition zones produced by antibiotics. (DOCX) [file pone.0102697.s002.docx]

**Table S1.** List of *Salmonella* strains used in this study and growth inhibition zones produced by antibiotics.

| Strains | Serotypes | Origin | ^a^Resistant  antibiotics | Diameter of clear zone (mm) | | | | | | | | |
| --- | --- | --- | --- | --- | --- | --- | --- | --- | --- | --- | --- | --- |
|  |  |  |  | AM  10µg | AMC  20µg | C  30µg | CF  30µg | NA  30µg | NOR  10µg | S  10µg | SXT  2µg | TIC  75µg |
| WS 1 | *S.* Gallinarum  ATCC 9184 | Chicken | AM, AMC,  C, S, TIC | <8 | 10 | 12 | 27 | >30 | >30 | 8 | 28 | 13 |
| WS 2 | *S.* Gallinarum | Chicken | CF, NA, NOR,  S, SXT | 27 | >30 | >30 | 8 | 12 | 11 | 8 | 10 | >30 |
| WS 3 | *S*. Gallinarum | Chicken | NA, S | >30 | >30 | 28 | >30 | 8 | >30 | 12 | 28 | >30 |
| WS 4 | *S.* Typhimurium | Cattle | - | 28 | >30 | 28 | 28 | >30 | >30 | >30 | >30 | >30 |
| WS 5 | *S.* Typhimurium | Pig | AM, AMC, S, TIC | <8 | 8 | >30 | >30 | >30 | >30 | <8 | 27 | 11 |
| WS 6 | *S.* Enteritidis | Chicken | - | 27 | >30 | 28 | >30 | >30 | >30 | >30 | >30 | >30 |
| WS7 | *S.* Typhi ATCC 19943 | Human | - | >30 | >30 | >30 | >30 | >30 | >30 | >30 | >30 | >30 |
| WS 8 | *S.* Paratyphi A | Human | - | >30 | >30 | >30 | >30 | >30 | >30 | >30 | >30 | >30 |
| WS 9 | *S.* Enteritidis | Chicken | - | >30 | >30 | >30 | >30 | >30 | >30 | >30 | >30 | >30 |
| WS10 | *S.* Enteritidis | Chicken | - | 28 | >30 | >30 | >30 | >30 | >30 | >30 | >30 | >30 |

AM, ampicillin; AMC, amoxicillin/clavulanic acid; C, chloramphenicol; CF, cephalothin; NA, nalidixic acid; NOR, norfloxacin; S, streptomycin; SXT, trimethoprim/sulfamethoxazole; TIC, ticarcillin. ^a^The antibiotics resistance was reflected in the diameter of clear zone (mm) values for AM (≤13mm), AMC (≤13mm), C (≤12mm), CF (≤13mm), NA (≤13mm), NOR (≤13mm), S (≤10mm), SXT (≤10mm), TIC (≤14mm) .
